# Contact Allergy—Emerging Allergens and Public Health Impact

**DOI:** 10.3390/ijerph17072404

**Published:** 2020-04-01

**Authors:** Wolfgang Uter, Thomas Werfel, Jean-Pierre Lepoittevin, Ian R. White

**Affiliations:** 1 Department of Medical Informatics, Biometry and Epidemiology, University of Erlangen/Nürnberg, 91054 Erlangen, Germany; 2 Department of Dermatology and Allergy, Division of Immunodermatology and Allergy Research, Hannover Medical School, 30625 Hannover, Germany; Werfel.Thomas@mh-hannover.de; 3 Laboratoire de Dermatochimie, University of Strasbourg, Institut Le Bel, 67081 Strasbourg, France; jplepoit@unistra.fr; 4 St. John’s Institute of Dermatology, Guy’s Hospital, London SE1 9RT, UK; ian.white@kcl.ac.uk

**Keywords:** allergic contact dermatitis, contact allergy, contact sensitisation, epidemiology, prevention, review

## Abstract

Contact allergy (sensitisation) and allergic contact dermatitis (ACD) resulting from it have a considerable public health impact. For the present review, all pertinent articles were systematically searched via Medline and Web of Science™; additionally, all available issues of the journals “Contact Dermatitis” and “Dermatitis” were manually searched, covering the years 2018–2019, thereby extending and re-focusing a previous similar review. New allergens, or previously described allergens found in a new exposure context or of other current importance, are described in sections according to substance classes, e.g., metals, preservatives, fragrances. As a common finding in many investigations, a lack of information on product composition has been noted, for instance, regarding a newly described allergen in canvas shoes (dimethylthiocarbamylbenzothiazole sulfide) and, most notably, absence of co-operation from manufacturers of glucose-monitoring devices and insulin pumps, respectively. These latter devices have been shown to cause severe ACD in a considerable number of diabetic patients caused by the liberation of isobornyl acrylate and N,N’-dimethylacrylamide, respectively, as demonstrated by an international collaboration between dermatologists and chemists. Improved and complete ingredient labelling for all types of products, and not just as we have with cosmetics at present (apart from full listing of fragrance substances) in Europe, must be put on the legislative agenda.

## 1. Introduction

Contact allergy is an acquired immunological alteration caused by skin, or occasional mucosal or systemic, contact to (generally) low molecular weight substances. Despite considerable efforts, it remains largely unclear and thus unpredictable as to who will, under the same conditions of exposure, develop contact allergy and who will not. However, some driving forces of acquisition of contact allergy are well known: (i) the hazard in terms of the sensitising potency of a substance, (ii) exposure conditions regarding dose/area, repeated and/or aggregated exposure to the substance resulting in sensitisation risk, and (iii) general susceptibility factors such as a compromised epidermal barrier, prevailing cutaneous inflammation, as well as the yet unidentified individual predisposing (susceptibility) factors. According to current epidemiological data from Europe, some 20% of the population are affected by contact allergy to one or more of the about 30 most important or common substances causing contact allergy [1,2].

Once sensitisation has occurred, each subsequent exposure to the sensitiser (contact allergen) above the individual elicitation threshold will lead to clinical disease in terms of allergic contact dermatitis (ACD). ACD can be severe and widespread, and may become chronic and disabling, particularly if the offending contact allergen is not identified; diverse obstacles exist before a correct diagnosis is achieved (see discussion). According to current knowledge, contact sensitisation cannot be “cured” and is considered to persist life-long. Therefore, allergen avoidance is the key to avoid relapses of ACD. Sufficient avoidance may be easier to achieve for some contact allergens than for others, and also depending on the individual intensity of sensitisation, which may vary by 2 or 3 orders of magnitude of eliciting dose/area [3]. It has been estimated that in Germany, 12.7% (95% confidence interval (CI): 11.5–14.0%) of the population has, at some point in their lifetime, suffered from ACD [4]. Hence, the public health impact of contact allergy and ACD, respectively, is considerable. Work-related ACD, with its associated individual and societal costs and losses in productivity, has additional impacts.

From a public health perspective, with a focus on prevention, the following topics are discussed in the sections of this paper: (i) hazard assessment (Section 4.2), (ii) risk management in terms of labelling and restrictions (examples in Section 3.3), (iii) secondary prevention based on adequate diagnosis and individual exposure assessment (Section 4.4). The present review intends to substantiate these more conceptual, general points and also to briefly update on new allergens or allergens encountered in new exposure contexts—both of which are important for clinicians to adapt their “diagnostic radars” to ever-changing conditions, ultimately to the benefit of their patients (Section 3). To this end, new evidence published in the years 2018 and 2019 has been systematically and additionally hand-searched. The scope of the present review, as presented in Section 3, follows up on a similar review covering the years 2016 and 2017 [5].

## 2. Methods

A systematic search using the search words and fields (“contact sensitisation” OR “contact allergy”) AND (“2018/01/01”[Date-Publication]: “3000”[Date-Publication]), “3000” meaning until the current day, was used in Medline, and the equivalent in Web of Science™ (WoS; core collection) mid-September 2019. The former yielded 231 hits, the latter, 285. Both search results were merged, and duplicates removed. The results were analysed on the level of title and abstract and either excluded, if not pertinent to the topic of the present review, or further processed by reviewing the full text. Altogether, *n* = 118 publications were included that had been found in both bibliographic resources, *n* = 23 found in Medline and *n* = 19 exclusively in WoS, thereby arriving at *n* = 160 citations reviewed on the fulltext level. The reasons for exclusion were the publication type being a review, editorial or meeting abstract (*n* = 74), basic science results not pertinent to the article’s topic (*n* = 36), diseases other than contact allergy/dermatitis in focus (*n* = 48), therapeutic drug studies (*n* = 9), obvious poor quality (*n* = 6) or lack of new evidence (*n* = 19, meaning no new, just confirmative, evidence in relation to publications before the study period), and pre-print status (*n* = 41), with overlap between the two bibliographic resources. The process was performed by a single investigator (WU), as duplicate/consensualised extraction of information and, particularly, assessment of bias of reports normally employed in the preparation of systematic reviews was not deemed necessary in the present, strictly descriptive context.

The main aim is to sensitively identify all relevant publications. To this end, all issues of the journals “Contact Dermatitis” (ISSN 0105-1873) and “Dermatitis” (ISSN 1710-3568), the two pertinent sub-specialty journals in the field, were additionally manually searched for references missed by the above literature searches. Moreover, in selected cases, important publications, mostly reviews, were also included even if published earlier. The following topics were considered out of scope for the present review: cutaneous adverse drug reactions, atopic dermatitis, and immediate-type hypersensitivity reactions, therapeutic studies, and photodermatitis.

## 3. Results

**Figure 1:**
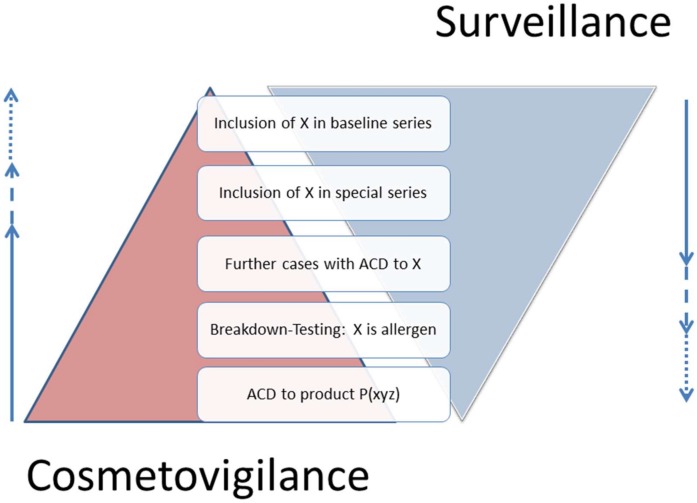
Suitable cosmetovigilance complements contact allergy surveillance. X represents any chemical substance (or perhaps natural mixture). P (xyz), any product containing the substances X, Y and Z (taken from [6]).

In the following sections, important new findings are presented grouped according to the type of allergens. It has to be noted that in several cases, these findings could be put under more than one heading, such as cobalt exposure via leather sofas, plant-derived material used as medicine and preservatives used in textiles or leather. As a general rule, the categorisation in the following sections is according to the nature of the allergen and not the context where it has been found, the only exception being allergens found in medical devices owing to the prominent role of this problem. Referring to the above list of examples, the first would be found under “metals”, the second under “plants” and the third under “preservatives”. Moreover, published results range from case reports—often the first indication of an emerging contact allergen—to systematic testing in consecutive patients, and the different underlying approaches, namely, vigilance vs. surveillance, are illustrated in Figure 1.

### 3.1. Metals

**Figure 2:**
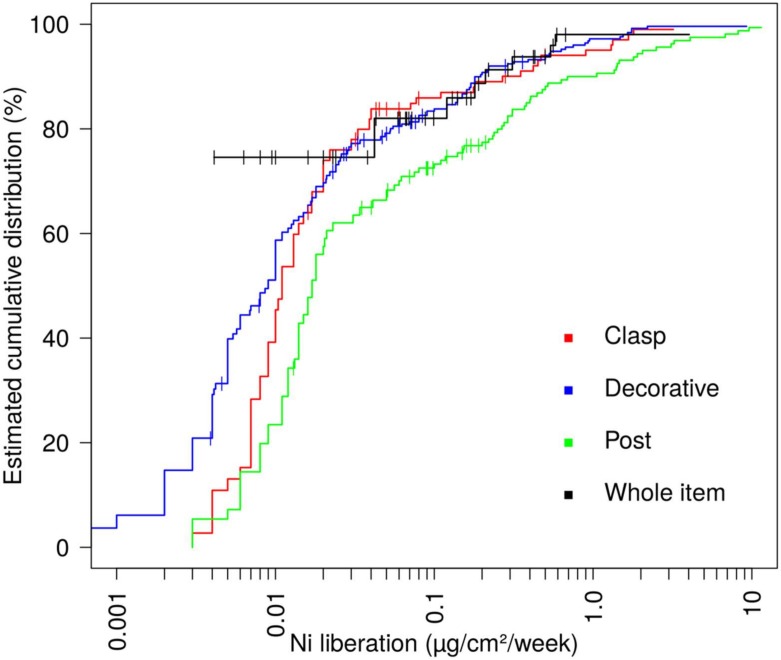
Distribution of measurements of nickel (Ni) release after immersion according to EN 1811:2011, stratified for four different parts. The curve represents the estimated cumulative distribution function of the measurements, as obtained from Kaplan–Meier analysis. Left-censored measurements, i.e., those with a missing measurement which has been replaced with the limit of quantification of the respective sample, found in 49.3% of all samples, are indicated by short vertical bars (taken from [11]).

**Nickel** regulation was implemented first in single Nordic countries and later by the EU Nickel Directive in 1994, coming into force in 2000 and into full force from 2001 onward [7]. Later, in 2009, the EU Nickel Directive was included in REACH, the EU Chemicals Regulation (EC) No 1907/2006, Annex XVII Section 27. Previously, costume jewellery has been the main source of nickel exposure resulting in sensitisation. A relatively recent population-based epidemiological study with patch testing a random sample of *n* = 3119 in five European countries found the lowest prevalence of nickel contact allergy (8.3%) in Sweden, whereas overall, it was 14.5%, and highest in Portugal (18.5%), supporting the benefit of long-standing regulation [8]. In Danish children patch tested during 2012–2016, a significant decrease of nickel allergy compared to 2003–2011 has been noted [9]. Piercings increase the risk of nickel allergy, ranging from “piercing ever, but not currently” (OR 3.86, 95% CI: 2.85–5.24) to “currently ≥ 3 piercings” (OR 5.58, 95% CI: 4.02–7.76). Even after many years of regulation [10], e.g., of earrings and other piercings, jewellery still constitutes a major source of nickel exposure. A German 2014 survey found that nickel release exceeded 0.35 μg/cm^2^ per week in 26 of 160 piercing posts (16.2%) and 0.88 0.35 μg/cm^2^ per week in 2–5.9% of other parts; these limit values correspond to the current ”pass” criteria [11]. The distribution of nickel release across different parts of the piercing jewellery is shown in Figure 2.

Toys, such as “fidget spinners”, may release nickel potentially sufficient to elicit ACD [12]. The wristwatch of her boyfriend regularly caused ACD in a 27-year-old woman when sitting at his side in the cinema [13]. Also, in an occupational context, nickel exposure (and ACD) may occur, for example, via embroidery needles [14] or in metal tools used in hairdressing [15], even though nickel-releasing items are prohibited by occupational hygiene regulations in Germany. As expected, nickel is an important occupational contact allergen in the USA, where nickel exposure is not regulated [16]. A structured approach to assessing items used by the patient may help identify important sources of exposure [17]. Spot testing using the dimethyl glyoxime (DMG) test, possibly using an electrochemical device for better standardisation [18], is indispensable for determining individual exposures. For the diagnosis of nickel allergy by patch testing, the standard nickel sulfate hexahydrate 5% in petrolatum (pet.) is usually sufficient, which is now also recommended in the USA, where traditionally 2.5% pet. is used, owing to a significantly higher sensitivity [19]. If doubtful or negative reactions result with 5% pet., despite a strong suspicion of nickel allergy, testing with nickel sulfate hexahydrate 30% and 15% aq. can be considered [20].

Cosmetics may contain nickel (e.g., as a contaminant of iron oxide pigments) as shown by two cases of ACD affecting the eyebrows due to an eyebrow pencil containing 8–9 mg/kg nickel [21]. A study including 45 nickel-sensitised individuals found elicitation by short patch test exposure—corresponding to the periods laid down in the ECHA guidance—in only a few of these [22]. However, it has been criticised that this exposure does not reflect real-life exposures [23]. Therefore, and in view of a vast array of other evidence, the current definitions of “nickel contact” are still regarded as valid. This is supported by the observation of considerable nickel deposition and stratum corneum penetration after short repeated contacts of 3 × 10 min [24].

Besides jewellery and similar items, sources of nickel or other metals may be hidden, as illustrated by a covertly located metallic nickel-containing retainer inducing allergic contact mucositis due to nickel allergy [25]. Openly worn, but still unexpected, pieces of a meteorite on a wedding ring have led to ACD in a nickel-sensitised 28-year-old male [26]. Military (or other) decorative pins, if coming in contact with skin, may cause ACD [27]. Using a novel approach (time of flight secondary ion mass spectrometry [ToF–SIMS]), nickel deposition in the stratum corneum and upper epidermis could be traced; this method is hoped to aid future toxicokinetic studies on allergen absorption and penetration [28].

**Cobalt**, patch tested as cobalt chloride hexahydrate 1% pet., is a “difficult” allergen, as (i) the clinical relevance of positive (allergic) reactions can only be established in a minority of positive patients [29] and (ii) metachronous reproducibility is quite limited [30]. Some cobalt release, assessed similarly to the EN1811:2011 + AC:2012 method used for nickel, has been identified from piercing posts and decorative parts of costume jewellery [11]. Dark shades of paints may rarely contain cobalt, but a recent large sample of USA paints did not find any cobalt by spot testing and X-ray fluorescence spectroscopy [31]. The hard metal industry is a well-known risk setting for cobalt exposure, which had been quantified by a Swedish study by sampling 40 workers with the acid wipe technique after 2 h of work. Analysis with inductively coupled plasma mass spectrometry (ICPMS) demonstrated median skin doses of 0.5–1 μg/cm^2^, with extreme values as high as 28 μg/cm^2^ [32]. Leather is an important source of cobalt exposure, as demonstrated by the analytical results of a convenience sample of leather shoes and gloves in Denmark, in which 7/43 shoes and 3/16 gloves contained >1% cobalt, unrelated to the colour of the leather [33]. Rarely, cobalt residues in machine oil may cause ACD [34].

After the regulation of hexavalent **chromium** (dichromate) in cement in the EU, contact allergy to chromium from this exposure has declined. This is not the case in countries without similar regulation, such as India, where dichromate levels in cement are still high [35]. Chromium-tanned leather has been tackled by another regulation, limiting the content of dichromate to <3 mg/kg leather (Commission Regulation (EU) No 301/2014 of 25 March 2014, amending Annex XVII to Regulation (EC) No 1907/2006); the mid-/long-term effect of this recent preventive step is still awaited. In Danish patients tested between 2002 and 2017, the prevalence declined significantly, and leather became the most commonly relevant exposure source (in 55% of patients at the end of the study period) [36]. Between 2005 and 2014 (i.e., before the above regulation), the prevalence of chromium allergy declined only very moderately, more in males than in females, in southern Sweden [37]. An Italian study analysed 29 tattoo inks purchased in Europe and the US for the presence of hexavalent chromium by ion chromatography and ICPMS. Cr(VI) was identified in 90% of samples, with levels of 0.22–4.09 mg/kg, i.e., well above the maximum level allowed [38]. A very interesting use test study involving 10 Cr-allergic subjects demonstrated that common levels of dichromate in the study leather bracelet worn in a controlled fashion were able to elicit ACD in 4 of these (while only 1 reacted to the leather sample in patch testing) [39]. Cr(III) has been found to be released in significantly higher amounts than Cr(VI) in a survey on leathers produced in Nicaraguan tanneries, and it was suggested—in view of the possible elicitation of ACD even by trivalent chromium, given sufficient exposure levels—that regulation should also focus on Cr(III) [40]. A case report of a 27-year-old US male with ankle (but not plantar) dermatitis pointed to regionally differing sensitivity to elicitation by dichromate: the total Cr content was lower at the ankle that in the leather sole (225 mg/kg vs. 268 mg/kg) [41]. Unfortunately, the analysis of total Cr does not reflect the speciation into tri- and hexavalent chromium relevant for sensitisation/elicitation. A peculiar clinical pattern of ACD in the lumbar region was observed in a 43-year-old Dutch scaffolder, who wore a tool holster belt made from leather, and was found allergic to chromium [42]. Similarly, the anatomical site of dermatitis in a 37-year-old London office worker commuting by car (that is, driving for prolonged periods) together with a positive reaction to potassium dichromate on patch testing quickly pointed to the leather-covered steering wheel and gearstick as his cause of problems [43]. The colorimetric 1,5-diphenylcarbazide “spot test” is available for the easy detection of hexavalent chromium. As recently demonstrated, false-positive results may, however, arise when testing aged trivalent chromium passivation layers, where no hexavalent chromium is detected with more accurate methods such as X-ray photoelectron spectroscopy [44].

**Palladium** can cause granulomas, e.g., in pierced earlobes, as in a 28-year-old Dutch female. Interestingly, the positive patch test reaction to palladium chloride 1% pet. also evolved into a persistent inflammatory reaction. By the use of ICPMS, a 3-fold higher palladium signal than in control biopsies was identified in the lesion [45]. In consecutively (routinely) patch-tested Spanish patients (*n* = 3678), palladium dichloride 2% pet. caused 8.6% positive reactions; only 26/316 positive patients were not also sensitised to nickel [46], confirming observations made elsewhere [47]. Patch test reactions to **vanadium** (tested as trichloride 1% pet.) are difficult to interpret; additional evidence for the role of vanadium contact allergy in the hand eczema of a 39-year-old steel manufacturing worker was provided by a controlled lymphocyte transformation test (LTT) [48]. A worker in a tin-smelting factory developed spreading lichenoid ACD and was found patch-test positive to tin 50% pet.; reduction of exposure by reducing heat and thereby fumes already led to significant improvement [49]. Strong evidence, even with a weak positive, late reactivity in patch testing for the role of **indium** as a cause of orofacial granulomatosis (following insertion of indium-containing dental crowns) has been identified in a Japanese patient; following removal/replacement of the crowns, the lesions improved [50].

If **aluminium** patch test chambers (e.g., Finn Chambers™) are used, positive reactions to all applied substances will quickly point to aluminium being the actual allergen [51]. A re-test using plastic chambers, and an aluminium salt for further confirmation, is indicated [52]. Use of 2% aluminium chloride hexahydrate aq. is more sensitive than metallic aluminium and recommended for children with deep cutaneous inflammatory nodules after vaccination with aluminium-containing vaccines [53,54]. In a French patch test study involving 97 children, using 2% aluminium chloride hexahydrate pet., 21.6% were found sensitised; 8/21 also reacting to metallic aluminium. All 5 children tested for persistent itching nodules were allergic, but also 11 without such history [55].

Metallic **gold** is a quasi-inert agent, while gold ions are highly reactive and capable of causing contact allergy. A 50-year-old female patient with a gold implant of 999.9 fineness in her upper lid (due to unilateral facial palsy) developed allergic lid dermatitis, which resolved after removal; the patch test was positive to gold sodium thiosulfate 0.5% and 2% [56]. In a similar case from Spain, a late cutaneous B-cell pseudolymphoma was observed in the upper eyelid [57], in another case, also from Spain, features of Langerhans cell histiocytosis were detected in a patch test reaction appearing 5 months after application of gold sodium thiosulfate 75 μg /cm^2^ [58]. This dose, applied with the TRUE Test™, is regarded as offering the best sensitivity in diagnosing gold allergy [59]. Patch testing to gold is often performed with gold sodium thiosulfate, yielding (i) positive test reactions in a considerable number of patients, and (ii) late-appearing reactions which may either represent active sensitisation or a low degree of sensitisation/reactivity [60,61]. According to a Bulgarian study, dental professionals (students, doctors and technicians) may have an increased risk of sensitisation to gold, palladium and other metals used for crowns [62].

**Titanium** is widely used in orthopaedic or dental implants. Patch testing for titanium contact allergy is difficult, as titanium dioxide usually does not cause positive reactions. In a Dutch study, various titanium salts were patch tested, giving a highly variable yield of positive reactions, of which 62% were deemed relevant by the authors, who suggest prospective studies to arrive at a definite conclusion regarding the utility and validity of titanium patch testing [63].

Hypersensitivity to metals as a cause of implant-related complications has been a subject of controversy. In a Swiss study, patients with implant-related complications or a positive history of contact allergy and planned total joint replacements referred for allergological investigation between 2004 and 2017 were retrospectively analysed. Among the 311 patients, a positive patch test reaction to a metal was seen in 64.4% of preoperative patients and in 54.6% of patients with implant-related complications. Common alloy metals such as cobalt, chromium and titanium gave positive reactions in up to 2.9% of patients with implant-related complications. None of the patients with skin changes had a positive patch test reaction to an implant metal. In conclusion, other factors, such as the type of replaced joint and mechanical stress, seem to be more relevant for implant-related complications. Sensitisation to metals or other materials seems to rarely play a role and is overestimated [64].

### 3.2. Fragrances

Screening for fragrance substance contact allergy in routine patch testing is achieved by the use of two fragrance mixes (I and II), including 8 and 5 single constituents, respectively. Furthermore, a natural mixture, *Myroxylon pereirae* resin, is routinely tested, containing a number of constituents to which exposure is also possible independent of this mixture, while other constituents are more characteristic for *Myroxylon pereirae*, and some constituents are shared with propolis (see under “woods, plants and plant products” below), as recently reviewed [65]. It has repeatedly been found that using these screening markers, only about half of all fragrance-allergic patients are diagnosed; hence some departments routinely test with all 26 fragrances currently needing to be labelled in the EU according to Annex III of EU Cosmetic Regulation 1223/2009 [66].

Probably the “hottest” topic in the field of fragrance contact allergy is the role of **terpene hydroperoxides** as autoxidation products of terpenes used as fragrances, and specifically, of limonene and linalool hydroperoxides (Lim-OOHs and Lin-OOHs, respectively). These have been demonstrated to be important contact allergens when patch tested (in consecutive patients) [66,67,68], in accordance with experimental results [69]. However, hitherto there is still uncertainty regarding the quantity and eliciting role of hydroperoxides present in cosmetic products patients have actually been using. At the same time, limonene and linalool are the fragrance substances most often used in cosmetic and consumer products [70,71]. From this background, a report from Spain of a 35-year-old female is interesting: in a dermatitis reminiscent of early cutaneous T-cell lymphoma, patch testing revealed contact allergy only to Lim-OOHs 0.3% pet. and her own cosmetic product. Avoidance of all limonene-containing products led to clearance of the skin after a few months, and a controlled re-exposure to a previously used, limonene-containing shampoo led to a relapse after a few weeks [72]. Axillary ACD developed in a 60-year-old non-atopic Swedish female following the use of a scented deodorant and resolved after switching to an unscented product. Patch testing was positive to Lin-OOHs 1% pet., which could be identified by HPLC in the culprit deodorant (8.4 ppm linalool-6-hydroperoxide and 5.6 ppm linalool-7-hydroperoxide); a subsequent re-introduction of the previously used deodorant in one armpit caused ACD after only 10 days on that side [73]. In a systematic approach, Bennike et al. studied the elicitation threshold and dose–response relationship of Lim-OOHs in 11 individuals with a positive or doubtful patch test reaction to standard Lim-OOHs 0.3% pet. by a 3-week double-blind vehicle-controlled repeated open application test (ROAT) study with a simulated fine fragrance containing Lim-OOHs at 1260, 420 and 140 ppm, equal to a dose/area per application of Lim-OOHs of 3.0, 0.99 and 0.33 μg/cm^2^. Among the 11 subjects allergic to Lim-OOHs, 11 (100%), 7 (64%), and 3 (27%), respectively, reacted to the applied doses. No reactions were seen in 17 healthy controls exposed to the highest dose (*p*  <  0.0001). It was concluded that contact allergy to Lim-OOHs is clinically relevant in patients with positive patch test reactions and even in some with a doubtful patch test reaction to Lim-OOHs 0.3% pet. [74].

As well as limonene and linalool hydroperoxides, oxidised geraniol is now also in focus. In a Swedish study with 1476 consecutive patients, 3% have shown positive reactions to oxidised geraniol 6% pet. and 8% to geraniol 11% pet., the higher test concentration also yielding 5% doubtful reactions [75]. Geraniol was found to be the most frequent allergen among the constituents of FM I in a Spanish multicentre study; of note, 230 of 19,588 patients tested were positive to fragrances not covered by the mixes [76]. Among these, tree moss (*Pseudevernia furfuracea* [L.] Zopf.), syn. *Evernia furfuracea* [L.] W. Mann, a lichen growing on conifers, is a frequent sensitiser. Previous studies have shown two subgroups of tree moss-allergic patients: a group sensitized to common allergens of tree and oak moss (*Evernia prunastri* [L] Ach.), and another group sensitised to colophonium-derived allergens, which may contaminate tree moss extract. In a Danish study, 22 of 632 tested patients (3.5%) had positive reactions to tree moss. Eight patients were sensitised to tree moss only (among fragrance allergens), and 75% had relevant reactions to colophonium. In conclusion, the prevalence of tree moss reactions was considered high enough to justify its inclusion in the baseline series. If tree moss is not included, patients with positive colophonium reactions should be informed of possible (false) cross-reactivity to tree moss to avoid this labelled fragrance allergen [77].

**Fragrance mix I** is still the most important screening mixture comprising seven chemicals and one natural extract (*Evernia prunastri*). Among its constituents, cinnamyl alcohol and the corresponding aldehyde, cinnamal, are important allergens. As not all patients reacting to cinnamyl alcohol are also positive to cinnamal; possible other haptens, considering either other metabolic activation pathways or cinnamyl alcohol being (partially) a prehapten, were investigated. On the one hand, studies on reconstructed human epidermis suggested that cinnamyl alcohol could be directly activated by a sulfotransferase to modify epidermal proteins [78]. This mechanism that would explain skin sensitisation is also supported by the clinical case of a worker who developed severe allergic contact dermatitis after accidental exposure to cinnamyl chloride. [79]. At patch testing, he was found positive to cinnamyl chloride but also to cinnamyl alcohol. Cinnamyl chloride would be expected to form the same protein adducts as the sulfate ester of cinnamyl alcohol, thus explaining the concomitant reaction to both cinnamyl derivatives. On the other hand, epoxycinnamyl alcohol and epoxycinnamal appeared not to be important when tested in 12 patients [80]. FM I contained in the TRUE Test™ system is significantly less sensitive than the pet.-based allergen, as repeatedly demonstrated, lately by the EDEN Fragrance Study [81], which needs to be taken into account when comparing test results obtained with different patch test systems. Moreover, all commercially available patch test preparations contain sorbitan sesquioleate (SSO) as an emulsifier. As this is an allergen, albeit relatively rare, in its own right, it should be tested along with FM I [82].

While in the United Kingdom fragrance allergy seems to be on the decline, this was not the case with regard to occupational contact dermatitis related to fragrances, which remained stable, hairdressers and therapists being the occupations with the highest incidence rates [83]. In contrast, according to a Danish follow-up study involving 1496 patients with occupational hand eczema or contact urticaria, fragrances only ranked after rubber additives, biocides, hairdressing chemicals, nickel and epoxy resins regarding the share of sensitised patients [84]. Whether in the professionally exposed or in consumers, the often heavy scenting of hair products may lead to ACD by fragrances, as illustrated by a 70-year-old Spanish patient who was sensitised to benzyl salicylate found in several of the products she had been using [85]. Fragrances, including essential oils, may be found in mouth washes and may cause allergic contact stomatitis, as in the case of a 68-year-old Danish patient who was diagnosed with “burning mouth syndrome” but, effectively, sensitised to eugenol in her mouth wash used daily [86]. Carvone (*L*-carvone), a mint flavour in spearmint oil, is considered a weak skin sensitizer, allergy being linked to oral/perioral signs and oral lichen planus. In the Malmö clinic, 147 out of 4221 referred patients had a positive patch test to carvone; 73% had oral signs and 57% had oral lichen [87]. A Spanish patient was found sensitised to cinnamal present in her denture fixative cream [88]. Electronic cigarettes are gaining increasing popularity and may cause nickel ACD [89]. Furthermore, the vaporised liquid may also cause ACD, as in the case from Portugal, where menthol was the culprit allergen [90]. Vaporisation is also achieved by diffusers filled with essential oils, which has been previously reported to cause ACD in the exposed; recently, a 7-year-old child has been described who developed airborne ACD related to Lin-OOHs (+++ reaction in patch testing) [91]. The volatility of fragrances inherent in their nature may lead to unexpected diffusion, as illustrated by a 82-year-old patient who deodorised his stoma bag by use of a scented spray. Although spray was applied to the inside of the bag, the skin underlying the stoma bag suffered from ACD to *Evernia prunastri* and limonene; both allergens verified by patch testing were labelled on the spray product [92].

In Europe, only (or, at least) 26 fragrances need to be labelled in cosmetic products. However, many more fragrances have been identified as established human allergens or are likely allergens based on experimental (mostly animal) data [93]. In addition to single, chemically defined substances, natural extracts in terms of essential oils are important allergens, as reviewed in the above-mentioned SCCS-Opinion, and their use is widespread, e.g., in 47% of respondents to a US convenience sample of State Fair attendees [94] or in Thai luxury spa therapists [95]. A recent result from Australia points to the relevance of lavender extracts (in that country), with 2.2% positive reactions to different lavender extracts [96], further supported by the report of 2 cases from Italy [97]. It is important to extend labelling for improving consumer safety, and it is also important to establish adequate patch test vehicles and concentrations. This has been achieved for three additional substances, namely, 2-methyl-3-(3,4-methylenediocyphenyl)-propanal, cyclamen aldehyde and hexyl salicylate. In the dose-finding study, the former two elicited clearly allergic patch test reactions and thus warrant further testing [98].

### 3.3. Preservatives

The incidence of ACD to **methylisothiazolinone (MI)** has been unprecedentedly high in Europe and other parts of the world. Regulation (and prior pre-emptive partial withdrawals from the market) have led, in the EU and those countries adopting its regulation, to a significant decline, for instance, from 5.97% in 2015 to 4.72% in 2016, and further to 2.96% in 2017, according to an EECDRG study [99]. The same study also identified a significant shift of currently clinically relevant products, that is, those products which elicited ACD leading to consultation for patch testing, from leave-on cosmetics to rinse-off cosmetics and household products. The latter product category has led to a significant increase in occupational ACD in cleaners [100]. In Australia, the use of MI in leave-on products was banned in October 2017, while 100 ppm was maintained as the concentration limit for rinse-off cosmetics. After a peak of MI contact allergy prevalence of 20.3% in consecutively tested patients in 2015, the prevalence decreased to 11.4% in 2017 [101]; presumably, pre-emptive changes and the withdrawal in leave-on products already had a considerable impact. In Istanbul/Turkey, the prevalence of MI sensitisation also decreased from 9.4% in 2016 to 5.6% in 2018 [102]. In Thailand, an epidemic of MI sensitisation was also observed [103]. Although Thailand followed the EU regulation of cosmetic MI exposure, a market survey indicated the prolonged presence of MCI/MI and MI preserved products, respectively, even after regulation [104]. Conversely, in the USA and Canada, no MI regulation is in effect, and the 2015/16 data analysis of the North American Contact Dermatitis Group found 13.4% of patients sensitised to MI [105].

The multitude of products containing MCI/MI or MI may lead to widespread eczema caused by different products applied to different body sites [106]. Occupational exposures need to be considered too, such as by wall paints. Ninety-two percent of these, according to a recent survey involving chemical analysis of 60 paints bought in five European countries, still contain MI with median levels between 30 and 100 ppm [107]. Similar results were seen in a sample of 47 US paints, of which 96% contained MI and 94% benzisothiazolinone [108]. Moreover, 19/38 consumer adhesives bought in the USA contained at least one isothiazolinone, mostly MI (44.7%; 4–133 ppm), MCI (31.6%; 7–27 ppm) and benzisothiazolinone (15.8%; 10–86 ppm) [109]. This is not only a safety concern for those working with wall paints and similar (occupationally or non-occupationally) but also for the considerable number of persons already sensitised to MI, mostly via (leave-on) cosmetics. These may suffer from airborne ACD by the emissions of freshly painted walls, in which MI is detectable for many weeks, leading to problems for the sensitised inhabitants [110,111,112], which may be protracted to almost one year [113]. While photoallergic contact dermatitis is not in the scope of this review, it has to be noted that several patients sensitised to MI displayed photo-aggravated dermatitis, both clinically, and in the patch test, where a positive, but weaker reaction than after additional UV exposure was noted [114]. The beneficial impact of an adequate diagnosis of contact allergy—in this case, to MI—is illustrated by a study evaluating the QoL before diagnosis and at 3-months follow-up after diagnosis of MI contact allergy, finding a highly significant improvement [115]. However, particularly with more long-term follow-up, relapses despite counselling are not infrequent and mostly related to hidden sources of MI, the lack of labelling on industrial products, the complexity of cosmetic labelling, and remembering the name of the allergen [116]. A Thai study found that widespread dermatitis, seen in 20.2% of 3201 patients, demonstrated that preservatives were the significant offending allergens, especially MCI/MI and MI alone [117].

Children have repeatedly been found to become sensitised to isothiazolinones (or occasionally other preservatives) when producing “home-made” playdough (“slime”) or using commercial products containing these preservatives [118,119,120,121,122]. Misuse of a glitter glue sold on a market as “artistic material” as carnival make-up had resulted in papular face ACD [123]. Solutions used in traditional, non-digital photographic development may contain MCI/MI and other isothiazolinones, and cause ACD [124]. Another special group to consider are workers in the production of these biocides and household, industrial or other products containing them owing to possible (accidental) contact with high concentrations [125,126], which may sensitise by one single exposure.

Other **isothiazolinones** are not permitted in cosmetics, at least in the EU, but can be found in numerous household and industrial products. Exposure may sometimes be quite unexpected; case reports illustrating this help creating awareness of such sources. Some examples of ACD to “unexpected isothiazolinones” include octylisothiazolinone in compression stockings [127] and a leather sofa [128], benzisothiazolinone in a continuous positive airway pressure mask liquid soap [129], undeclared presence of MI in a face mask, probably due to preservation of raw materials [130], periocular contact allergy from spectacle frames cleaned with MI-containing household detergents [131], preservation of liquid “flower food” by MCI/MI and octylisothiazolinone [132], virtual camouflage of MCI/MI by use of a brand name (Acticide™ MV) in the ingredient information of a hospital hand-soap [133], and possibly triggering of oral pemphigus vulgaris in a 79-year-old Italian patient sensitised to MI by a mouthwash containing MI [134].

**Formaldehyde** is rarely used as such in cosmetics nowadays, while a number of formaldehyde-releasing preservatives are commonly found. Patients who are allergic to formaldehyde are at risk—to a varying extent depending on the type of releaser and factors such as pH—of ACD caused by such releasers, as demonstrated by two young female patients [135]; it is thus crucial to supply these patients with a list of such releasers. Using the chromotropic acid method, formaldehyde can easily be detected, also in personal care products (e.g., in 4 of 46 products in that study) where it, or where formaldehyde releasers, are not declared [136]. Most patients are exposed to more than one product containing formaldehyde releasers, and although contact allergy to each of these is below 1% in consecutive patients [137], this adds up to a considerable burden of morbidity. An unexpected source of formaldehyde, eliciting ACD of the hands in a sensitised subject, has been described from the USA, where Mr. Clean Bliss™ gloves were found to release formaldehyde using the chromotropic acid method [138]. Among a sample of 28 US nail polishes advertised to be “formaldehyde-free”, four were found to actually contain formaldehyde [139]. It can be concluded that product labelled ingredient lists and available information are often inadequate to confirm the potential for formaldehyde exposure and chemical-based (chromotropic acid) spot test kits may have utility for identification of potential formaldehyde exposure from cosmetic products [140].

**Sodium metabisulfite** (SMBI) is not an infrequent allergen and hence a candidate for inclusion in the European baseline series [141]. Clinical relevance, that is, causative exposures, are not always clear. Some case reports do illustrate SMBI may be an important allergen: Systemic allergic dermatitis followed an enema containing SMBI [142]; in another case localised erythema and swelling after injection of a local anaesthetic preserved with SMBI was first seen, followed by a generalised reaction after injection of 1% xylocaine with adrenaline (again preserved with SMBI) explained by an extreme positive reaction to SMBI 1% pet. when patch testing this patient [143]; a French supermarket employee exposed to sulfite-containing seafood developed ACD in terms of face dermatitis [144]; in a US patient, contact allergy to SMBI had contributed to granulomatous cheilitis by exposure to a daily pre-packaged deli sandwich [145]. SMBI may be an important allergen in patients with anogenital dermatoses [146].

**Parabens** are very weak allergens, also considering their use in cosmetics and other products, which was, at least in the past, extensive, with a sensitisation prevalence in consecutively tested patients of around 1% or less [147]. The case of a local anaesthetic-medicated plaster for post-herpetic neuralgia in a 50-year-old patient, who, after 4 months of daily treatment, developed ACD to the plaster, and was found allergic to the parabens included in it, illustrates the so-called paraben paradox: despite ACD to parabens and a positive patch test to these (and a sample of the plaster), the patient tolerated paraben exposure in various cosmetics [148]. **Thiomersal** (thimerosal), a mercury-containing compound, was withdrawn from many products, including vaccines which, in the past, had caused a considerable number of non-relevant “positive” patch test reactions. Rare exposures do exist and may cause ACD, as in a 84-year-old patient with dermatitis of his ear canal due to a thiomersal-containing ear medicament [149]. The quaternary ammonium compound **benzalkonium chloride** is a widely used preservative or antiseptic additive. Although considered a weak allergen, prolonged exposure, particularly in case of a compromised epidermal barrier, may result in ACD, shown in a case of ACD to a bandage impregnated with this compound [150]. In three patients with ACD to different leather products (shoes, car seat, belt), their contact allergy could be attributed to **2-(thiocyanomethylthio)benzothiazole** used as a fungicide, thanks to investigative efforts to elucidate the nature of the allergen [151].

Several of the preservatives routinely patch tested are dissolved in water, as it is not possible to incorporate these in petrolatum, the preferred vehicle, namely, MCI/MI (tested 0.01% or 0.02%), MI (tested 0.05–0.2%) and formaldehyde (tested 1% or 2%). This creates some technical problems concerning stability and dosing, which are not in the focus of this article. As one similar example, it has been shown by duplicate testing of MCI/MI 0.01% aq. and MCI/MI, as included in the TRUE Test™, a pre-loaded patch test system, that the sensitivity of the latter system was considerably higher [152], which needs to be considered when comparing results obtained with different test systems.

### 3.4. Plastic Monomers and Rubber

Recent years have seen an increasing number of publications on yet another contact allergy epidemic similar to the MI epidemic; not to a novel, newly introduced substance, but to a group of allergens well-known from different exposure contexts—**acrylates and methacrylates**, namely, esters of acrylic and methacrylic acid, respectively. These have been used for decades in bone cement and in restorative dental materials, exposing dentists and dental technicians [153], and in the industry as gluing and sealing material. Currently, sensitising exposures to hydroxyethyl methacryalate (INCI name: HEMA) in artificial nails are affecting nail technicians and customers alike. An EECDRG study reported 136 cases of ACD caused by nail acrylates diagnosed by aimed testing; this corresponded to 67% of all cases of (meth)acrylate allergy in the period 2013–2015 [154]. The main allergens in this study included 2-hydroxyethy methacrylate (91.9% pos.), hydroxypropyl methacrylate (83.2% pos.) and ethylene glycol dimethacrylate (69.2% pos.). Routine patch testing with 2-HEMA in Italy identified 1.5% positive reactions in the 4025 patients tested [155]. Besides nail technicians in the numerous nail salons [156,157,158], home use of kits either for artificial nails or “long-lasting nail polish” offer other opportunities for sensitisation [159]. The upward trend regarding (meth)acrylate-related ACD is mirrored by a downward trend of sensitisation to tosylamide/formaldehyde resin, a constituent of “classical” nail polish [160]. Sources of exposure and, thereby, sensitisation and/or elicitation of ACD to (meth)acrylates may sometimes be unexpected. This is illustrated by a 42-year-old Japanese patient who had acquired contact allergy to (meth)acrylates during the hand-crafting of plastic jewellery and later suffered from severe facial ACD following application of a face mask which was found to contain 2-hydroxyethyl acrylate [161]. Previous sensitisation may generally cause unexpected problems, as illustrated by a 58-year-old German female who had suffered from ACD to acrylic nails a long time before a dental composite filling; she developed erosive allergic contact stomatitis under the picture of lichen planus, and later consulted before arthroplasty, potentially also involving exposure to (meth)acrylates [162]. Even a “classical” exposure by artificial nails may cause aberrant ACD, leading diagnosis on wrong tracks, like suspected lupus erythematosus in a 22-year-old Irish female [163]. Protective nitrile gloves are rapidly (within <30 min) penetrated by 2-HEMA, as indicated by a positive patch test through a layer of glove material [164].

**Epoxy resin** systems are another group of plastic monomers with ever-increasing importance. The baseline series only includes one resin, presumably the most important one: diglycidyl ether of bisphenol A (DGEBA). Other resins, such as DGEBF (bisphenol F-based resin), reactive diluents, or hardeners [165,166], are also important allergens. Correspondingly, many (occupational) contact allergies may be missed if relying solely on commercially available test allergens [167]. Awareness of workers is often underdeveloped, for instance, concerning contact with contaminated surfaces such as benches, clothing and tools [168]. Epoxy resin systems are known to cause face dermatitis [169] either via airborne contact or accidental exposure, e.g., in painters who are often sensitised to these (and to isothiazolinones which may also cause airborne ACD) [170].

The (usually occupational) use of plastic resin monomers calls for diligent use of personal protective or other protective equipment. Accidental spills may expose a worker to high concentrations of reactive monomers, which may lead to immediate sensitisation by such a single exposure to **diisocyanates** [171]. It has repeatedly been noted that, while diisocyanates are too unstable to be patch tested, the corresponding amines are a valuable screening tool, for instance, diaminodiphenyl methane, as in a recent case series with occupational ACD related to polyurethane foam from France [172].

***p*-*tert*-Butylphenol formaldehyde resin** exists in a number of varieties; the resin included in the (European) baseline series is one of these and is of importance. In the case of a 16-year-old Japanese goalkeeper with chronic hand eczema, different phenol compounds were detected—which did cross-react with above-mentioned routinely tested resin—in five pairs of goalkeeper’s gloves used by the patient [173]. A Finnish 44-year-old food packaging industry worker developed contact dermatitis of the wrists when operating two machines spray-coating the interior of beverage cans; the patient was diagnosed as having ACD to the phenol formaldehyde resin used for that purpose [174].

As well as plastic monomers, including residual monomers after incomplete curing, additives may cause contact allergy. The bright orange colour “**Solvent Orange 60**” which is, e.g., found in orange/yellow traffic lights, is also used in spectacle frames and may cause contact allergy in the wearer, or in those producing coloured plastic articles [175,176]. UV-absorbers are often added to plastic materials to prevent degradation. ACD on three different anatomical sites by three different products, all due to **drometrizole**, to which the 27-year-old female was found sensitised, has been described, namely in the nosepads of sunglasses, rubber straps of sandals, and a rubber wristwatch strap [177].

Contact allergy to accelerators used in natural or synthetic rubber gloves is not uncommon, especially in the healthcare sector. In a Swedish cross-sectional study of 311 health care workers with hand eczema, 6% were sensitised to rubber compounds, compared to 1% in those without hand eczema [178]. For those sensitised to **thiurams or dithiocarbamates** (the corresponding derivatives forming a redox pair and thus cross-reacting allergens), a switch to gloves containing only benzothiazoles is a theoretical option, but in view of poorly declared glove constituents, this approach entails the risk of failure. In this situation, the use of “accelerator-free” elastomer gloves, which are becoming increasingly available, is a viable alternative, as demonstrated in a French study involving nine healthcare workers with hand eczema [179]. In a person sensitised to tetraethylthiuram disulfide (TETD; disulfiram), e.g., by rubber gloves, systemic allergic dermatitis can ensue if this compound is taken for treatment of alcoholism with Antabuse™ [180]. A modern cause of thiuram ACD was related to the use of a rubber cell phone case in a patient with known thiuram allergy [181]. An accelerator not related to (if frequently used in conjunction with) thiurams, dithiocarbamates and benzothiazoles is **1,3-diphenylguanidine** (DPG), which is increasingly being used [182]. An experimental study found that the use of an alcoholic hand disinfectant before donning polyisoprene gloves containing DPG increased the amount of DPG recovered from the hands; using artificial sweat for comparison, 84% of the DPG was released within 10 min of wearing [183]. In addition, surgical sponges used for scrubbing before donning gloves may also contain accelerators and contribute to occupational ACD of the hands, as in a US surgeon sensitised to DPG [184]. In a Belgian study including 44 healthcare workers, 86% reacted positively to DPG, emphasizing the importance of this allergen [185]. Besides delayed-type allergy to rubber ingredients, also natural rubber latex (NRL) had sometimes been claimed to cause not only immediate, but also delayed-type hypersensitivity. Illustrating an important caveat, a carefully documented case from Italy demonstrates that the NRL extracts or suspensions used for patch testing are very likely preserved, e.g., with thiurams. Hence, in case of pre-existing contact allergy to thiurams, as in the patient presented, positive reactions to patch tested NRL cannot be interpreted as contact allergy to NRL, but represent “false cross-reactivity” [186].

### 3.5. Medicines (Active Principles and Excipients) and Cosmetics

Contact allergy to **corticosteroids** seems a paradoxical phenomenon, given the immunosuppressive effect of this class of drugs, but is well proven. The EBS contains two corticosteroids assumed to cover different antigenetic classes, namely, budesonide and tixocortol pivalate. A Spanish multicentre study involving 3699 consecutively patch tested patients added six other corticosteroids (methylprednisolone aceponate, mometasone furoate, prednicarbate, clobetasol propionate, betamethasone 17-valerate, and betamethasone 17,21-dipropionate). Overall, 1.46% (*n* = 54) showed a positive reaction to at least one of the altogether eight corticosteroids and, among these, 39 to one of the six additional corticosteroids. Interestingly, 24 of those 39 were not positive to any of the two screening markers, and, hence, contact allergy would have been missed when relying solely on these [187]. Still, no matter how extensive the set of screening corticosteroids, an individual diagnosis will usually have to include those substances not tolerated according to the patient’s history, and ideally also skin and provocation tests (in case of systemically administered derivatives) of putatively not cross-reacting substances for confirmation of a safe alternative [188].

**Benzocaine** is a local anaesthetic used, e.g., in throat lozenges; exposure can be unexpected, as in the case of a 26-year-old man with severe balanitis after using a latex condom equipped with benzocaine to prolong endurance [189]. In addition, other local anaesthetics such as tetracaine and cinchocaine may cause severe ACD as in a 56-year-old Portuguese man, following use of anti-haemorrhoidal ointments [190]. Some drugs are used both internally and externally, including diltiazem for topical treatment of anal fissures. This application has caused perianal ACD within just a few days [191]. **Opioids** have been described to cause occupational ACD, most recently in terms of a case series from Australia, where 11 individuals were diagnosed with occupational allergic contact dermatitis caused by opioids, with seven reacting to thebaine, five to morphine, four to norhydroxymorphinone, two to codeine, and two to oripavine [192].

More and more patients consult the internet for information on diagnosis and possibly treatment of their skin condition. The fact that self-diagnosis and subsequent treatment may not always be correct and beneficial, respectively, is illustrated by the case of a 32-year-old Italian patient who presented with severe eczema of his groins and scrotum after self-treatment of a presumed infection with an acne medication containing **benzoyl peroxide** (BPO). No matter which skin changes had initially prompted this treatment, he had acquired contact allergy to BPO with extreme positive reactions upon patch testing [193]. This observation is interesting beyond the actual case, as it is indicative of a presumably increasing problematic trend attending doctors need to be aware of.

**Calcipotriol**, a vitamin D3 analogue used for topical treatment of psoriasis, can rarely cause contact allergy; this is illustrated by the fact that in the highly specialised Leuven department, between 2004 and 2016, only six sensitised patients, among these an 11-year-old girl, were diagnosed by patch testing with 2 or 10 μg/mL in isopropanol [194]. In Brazil, **ketoconazole** is the only topical antifungal available in the national public health system, which may explain why contact allergy to ketoconazole 1% pet. was seen in 78 of 749 patients patch tested in São Paulo between 2010–2017 [195]. Some decades ago, **tioconazole** was found to be a significant contact allergen; in 2019, a new series of 8 patients with onychomycosis treated with 28% tioconazole solutions was reported from Spain, with patch tests using tioconazole 1% pet. being positive [196]. Additionally, undecylenic acid, which is also contained in the above nail solutions (but tested negative in the 2 patients tested with it) may be a cause of ACD from nail solutions [197]. **Amorolfine**, another antifungal used to treat onychomycosis topically in a lacquer, is capable of causing ACD [198]. ACD caused by **dexpanthenol** is considered rare; however, owing to the broad use and thus exposure, contact allergy may not be so rare, e.g., as seen in 1.2% of 2171 patients tested in Coimbra, Portugal [199]. **Timolol** is a well-known cause of ACD in a proportion of patients when used topically for anti-glaucoma therapy; not surprisingly, also the use of this (and other) beta-blocker for the treatment of infantile haemangioma may cause ACD [200].

*β***-Lactam antibiotics** are well-known causes of cutaneous adverse drug reactions not considered in this review. Healthcare workers preparing tables, injections or infusions of these antibiotics are at a high risk of becoming sensitised, not only in terms of immediate hypersensitivity, but also contact allergy leading to ACD, as shown by a case series from Portugal [201]. Employees in the production of drugs are a well-known risk group for skin (or other, immediate type) sensitisation, partly to finished drugs, partly to precursors. An apparently very rare case of ACD due to hydroxychloroquine sulfate in a 42-year-old production worker has been reported from Spain [202]. **Clindamycin** is used both as systemic drug and topically for acne or hidradenitis suppurative. A 58-year-old Italian patient with the latter diagnosis developed ACD to clindamycin gel; sensitisation was confirmed by a positive result to clindamycin hydrochloride 1% pet [203]. Ophthalmic preparations are a common source of exposure to topical antibiotics; consequently, these—mainly aminoglycosides—are relatively common causes of periorbital or lid ACD [204]. **Mupirocin** is a topically used antibiotic and a rare allergen, but has been reported to have caused severe ACD in a polysensitised French patient [205]. **Paromomycin** is used worldwide for the topical and systemic treatment of visceral, mucocutaneous and cutaneous leishmaniasis, and capable of causing ACD following topical application [206]. Before the treatment of HIV infection with **abacavir**, patients have to undergo genetic screening for the HLA-B*5701 variant as this genotype is associated with a high incidence of severe cutaneous adverse drug reactions. Based on two affected workers in the production of this drug, it was confirmed that also those topically exposed should be screened [207]. Patients treating their pets with various medications may develop “consort dermatitis”, e.g., to **tylosine** in a medicated powder for a dog [208].

Plant-derived cosmetic constituents are increasingly popular, perhaps owing to a perceived characteristic of being natural and thus safe. In reality, many chemically active substances are found in plants that are—partly strong—contact allergens (see the section on plants). Sometimes only the use of an extract in a cosmetic reveals sensitisation risk, as for *Scutellaria baicalensis* root extract [209].

A case of mild angular cheilitis, which could have been mistaken as a “minimal sign” of atopic eczema, was actually due to using a toothpaste with **stannuous fluoride**; positive patch test reactions were seen to the toothpaste as well as tin 50% pet., which is otherwise an extremely rare allergen [210]. Both children and adult patients with difficult-to-treat atopic dermatitis have high prevalences of concomitant allergic contact dermatitis and are frequently polysensitized [211], hence patch testing to identify contact allergy and secondary ACD to active ingredients or excipients is very important.

Excipients (matrix constituents) of medicines are mostly the same emulsifiers, stabilisers (and fragrances and biocides, see above) as used in cosmetic products and can, therefore, be viewed together. The only emulsifier included in the EBS [141] (and most other baseline series throughout the world) is lanolin alcohol. Both **lanolin alcohol** (wool alcohols) and its derivative **Amerchol™ L101** were tested in 9577 consecutive Danish patients from 2004 to 2015, and a massive increase in positive reactions from 0.45% to 1.81% was noted [212]. In contrast, in another study from central Europe with 82,251 patients, no significant trend was seen between 2006 and 2016 [213]. In a study from Amsterdam, the addition of Amerchol L101 significantly increased the yield of positive reactions [214]. **Cetearyl alcohol**, a mixture of cetyl and stearyl alcohol, is widely used as an emulsifier and an occasional allergen [215]. Various derivatives similar to lanolin alcohols exist, some of which were identified as contact allergens, such as cetearyl isononanoate [216]. Low-molecular weight polyethylene glycol is widely used in Turkey and has been found to be linked with nitrofurazone sensitisation, but has also been relevant when used in minoxidil solutions, and antiherpetic and corticosteroid creams [217]. **Alkyl glucosides** are widely used in cosmetics and other products, including a foam wound dressing, which caused ACD in a 70-year-old Belgian female [218], and several wound care and operative products causing ACD in 2 US patients [219]. As one such derivative, lauryl glucoside has been found a currently relevant contact allergen in 3 of 264 French patients with eyelid dermatitis [220]. In another patient from Belgium, arachidyl glucoside, tested positive at 5% pet. (+) and 10% pet. (++), has been identified as culprit allergen [221]. In a retrospective analysis of all patients patch tested with either a cosmetic series that includes five alkyl glucosides (decyl glucoside, lauryl glucoside, coco glucoside, cetearyl glucoside, and caprylyl/capryl glucoside) or a specific alkyl glucoside series from November 2013 to April 2017 in two UK centres, 5775 patients were included. Twenty-nine (1.04%) of the 2796 patients tested with the cosmetic/alkyl glucoside series had a positive patch test reaction to at least one of the alkyl glucosides, 23 (79.3%) patients were sensitized to multiple alkyl glucosides [222]. **Cocamide DEA** (diethanolamide) is a frequently used foam booster in cosmetics and includes a mixture of coconut fatty acids of various lengths, mostly between 8 and 18 carbons, including almost 50% lauramide DEA. Therefore, cross-reactions (including elicitation of ACD) are likely and demonstrated by a study from the USA [223]. The skin conditioning and antioxidant agent **3-*o*-ethyl ascorbic acid** has repeatedly been identified as a contact allergen, as in a recent case from Montpellier/France, where no cross-reactivity to ascorbic acid (vitamin C) was observed [224]. The UV-filter **ethylhexyl salicylate** is an uncommon allergen but may be occasionally relevant and possibly cross-reactive with benzyl salicylate, a fragrance compound [225].

Plant-derived constituents of cosmetic products and topical medicines may also cause ACD, for example, severe cheilitis in a 13-year-old boy due to the use of a lip balm containing **castor oil** (*Ricinus communis* seed oil). After a positive screening patch test with the balm “as is”, castor oil was tested 20% pet., provided by the manufacturer in product use concentration, and caused a ++ positive reaction [226].

In addition to (non-)oxidative hair dyes covered in the section “clothing, leather and dyes”, hair cosmetics contain preservatives and fragrances (also covered elsewhere) and, in case of permanent waving or relaxing agents, substances capable of temporarily splitting the keratin disulfide bonds. Besides the “classical” agent ammonium thioglycolate, **cysteamine hydrochloride** has been increasingly used and reported as an allergen in 7/17 Japanese hairdressers tested in one department (positive to 0.5% and 1% pet.) [227].

### 3.6. Medical Devices

Medical devices cover a wide range of medically used products, from band aids (adhesive plasters) to highly complex machines [228]. In the EU, a new Medical Devices Regulation has been in force since 2018: Regulation (EU) 2017/745 of the European Parliament and of the Council of 5 April 2017 on medical devices. Unfortunately, essential aspects such as obligations of manufacturers to co-operate in the work-up of adverse reactions to medical devices and full product ingredient/content information are not implemented. Therefore, it was only thanks to the combined investigative efforts of dermatologists and chemists that ACD to **glucose sensors**, **insulin pumps**, and similar equipment could be related to certain allergens, namely isobornyl acrylate [229,230,231,232,233,234,235] and *N*,*N*’-dimethylacrylamide [236]. These adhesive monomers apparently leached from the plastic encasing onto the outer surface of the adhesive (which originally did not contain these chemicals) and sensitised patients, owing to the prolonged exposure to the devices of several days to few weeks at a time. Independently, the adhesive itself may also cause ACD, including a well-known adhesive compound (colophonium), as illustrated by two cases from Italy [237]. Alternative products for patients sensitised to isobornyl acrylate have been evaluated, such as the Dexcom™ glucose monitoring system [238,239]. Barrier techniques are sometimes used, such as with film-forming agents [240] or hydrocolloid dressings [241], with variable success. The repeated observation of significant co-sensitizations between sesquiterpene lactones (SL mix) on the one hand, and the glucose sensor FreeStyle Libre™ and/or isobornyl acrylate on the other hand, has prompted chemical analysis. No evidence of the presence of SLs was found via GC–MS analysis. Cross-reactions between them seem improbable, but possibly, a common precursor for both, such as camphene, may exist [242].

Several modern medical dressing and **adhesives** contain various acrylates and methacrylates and may thereby cause ACD [243]. In a study from Reims/France, about half of all 73 patients with chronic leg ulcers and surrounding contact dermatitis were sensitised to modern dressings (mostly hydrocolloids and hydrogels); the rate of sensitization increased with the length of the presence of chronic leg ulcers [244]. ACD has also reported following the application of electrocardiogram (ECG) electrodes, where sensitisation to acrylic acid (0.1% pet.) but not to (meth)acrylates or cyanoacrylates was observed [245]. Products for stoma care include stoma bags with adhesives and products intending to alleviate irritation by the adhesives. Gantrez™ ES-425 is a common component of adhesive pastes, and 10 out of 13 patients tested to Gantrez™ ES-425 had a positive reaction [246]. A common problem in this and similar studies is the lack of complete information on the ingredients to identify the culprit allergen and enable the patient to avoid contact with other products containing the allergen in the future (see discussion). **Polyurethane plastics** are less important in terms of exposure to monomers (unlike in the building trade or plastic production) but may cause ACD when in prolonged contact with the skin, owing to the liberation of residual monomers. This has been shown in a 36-year old man with cystic fibrosis who had received prolonged courses of i.v. antibiotics and developed ACD beneath a polyurethane catheter. Patch testing was both positive to this tested “as is” and to diaminodiphenylmethane [247]. Cyanoacrylates are used as **surgical glues**, e.g., Dermabond™, and may cause ACD surrounding the site of an excision or wound repair [248]. In a review of 38 patients of a history of ACD to such surgical glues, mostly Dermabond™, found that just using ethyl cyanoacrylate 10% pet., which is available as commercial patch test allergen, only detected 29% of the cases and the addition of octyl cyanoacrylate 10% pet. increased detection rate to 50%. Patch testing glues “as is” and following scarification of the epidermis further increased test sensitivity [249]. Another cyanoacrylate, n-butyl-2-cyanoacrylate, under the brand name VenaSeal™ (Medtronic, Minneapolis, Minnesota), is used for sclerosing therapy of varicose veins and has caused ACD of the skin covering the sclerosed vein in a 45-year-old female patient [250].

### 3.7. Clothing, Leather, and Dyes

Chromium-tanned leather is a well-known source of ACD to chromium, see above under “Metals”. A case of foot dermatitis related to the use of Sperry Top Sider™ canvas sneakers in an 18-year-old Dutch female as well as a large case series involving 18 young females (age 14–22 years) in Belgium suffering the same type of dermatitis after wearing these brand shoes were reported in which there was no leather contact [251,252]. Strikingly, all patients reacted to the screening allergen thiuram mix 1% pet. in the baseline series and samples of their own shoes, partly extracts and thin layer chromatograms thereof, and finally to a compound identified by HPLC analysis, namely **dimethylthiocarbamylbenzothiazole sulfide** (DMTBS). Several patients reacted, in a dilution series of DMTBS, to a ≥ 0.001% concentration in acetone, pointing to a strong sensitising potency of this compound. DMTBS seemed to have been formed from two rubber ingredients as the result of the vulcanisation process [252]. Of note, no ingredient information had been available from the manufacturer’s side. Hyperkeratotic plantar ACD has been reported in a 74-year-old Japanese wearing plastic sandals with Tinuvin™ 770, a hindered amine light stabilizer identified as culprit allergen [253]. **Acetophenone azine** has previously been described as an allergen in shin pads and similar sports protective equipment; recently, another case from the UK has been published [254] as well as from France [255].

The reactive chemicals in **oxidative hair dyes** are important allergens, while the toxicological profile in terms of sensitisation hazard of non-oxidative dye components is less well investigated [256]. A study of 4314 patients tested with *p*-phenylenediamine (PPD) 1% pet. revealed an increased risk associated with previous hair dyeing (OR 6.0, 95% CI: 3.9–9.4), and, to a lesser extent, previous henna tattoo (OR 2.4, 95% CI: 1.5–3.7) and being a hairdresser (OR 2.1, 95% CI: 1.3–3.2) [257]. The polymorphism of *N*-acetyltransferase entailing slow or intermediate acetylation seems to be a risk factor for PPD sensitisation [258]. Not only hair colours, but also eyebrow and beard colours are used, respectively, and may cause ACD of the latter region [259,260]. Sometimes it is not the person actually applying hair dye who is affected, but his/her (dancing) partner in terms of so-called consort dermatitis [261,262]. Beyond the common sensitisers, including *p*-phenylenediamine (PPD) [263] and toluene-2,5-diamine, “rare” causes, e.g., by some couplers are occasionally reported, e.g., ACD to 1-napthol [264]. Derivatives of PPD have been created with the aim of attenuating sensitising potential (and possibly cross-reactivity with PPD). One such derivative is 2-methoxymethyl-*p*-phenylenediamine, which, according to an open use test study with 25 PPD-allergic volunteers, indeed only partially cross-reacts [265].

Textile dyes include many so-called disperse dyes, which partly cross-react with PPD (hence PPD can be used as marker allergen for these dyes) and partly do not cross-react, such as Disperse Blue 106 and 124. In an occupational case from Tunisia, severe ACD under the clinical picture of acute generalised exanthematous pustulosis had developed with sensitisation to Disperse Red 3, Disperse Blue 3, *p*-aminophenol, *p*-aminoazobenzene and the mix of Disperse Blue 106/124 proven by patch testing [266].

Pigments used in **tattoos** are a health concern, including contact allergy as a toxicological endpoint. A comparison of the skin sensitisation potential of three red and two black tattoo inks, using interleukin-18 as a biomarker in a reconstructed human skin model, yielded evidence of inflammatory response triggered by these, and also of a sensitisation potential of two of these [267]. Some pigments contain metals, e.g., as oxide, and thus potentially other metals as contaminants. In a patient who developed skin allergy in a green tattoo, nickel allergy was observed, and nickel found (among, e.g., iron, titanium and chromium) in the analysis of the patient’s affected tissue. Unfortunately, the question of whether nickel was the only allergen remained unclear [268].

### 3.8. Woods, Plants and Plant-Derived Materials

Generally, some plant constituents which are important contact allergens are available as pure substances, such as primin (from *Primula obconica*; now deprecated owing to predominance of primin-free cultivars [141]) and the three **sesquiterpene lactones** included in the respective mix (SL mix). In view of the multitude of plant constituents beyond these patch test preparations, it is often necessary to test with extracts, the composition of which may vary with the season of harvest, geographic region, chemotype, that is, the (epi)genetically determined and varying spectrum of plant substances of plants belonging to one species on morphological grounds, and other factors, making standardisation difficult. A large multicentre study from the IVDK including 1492 cooks, 851 florists and 118,358 other occupations found an increased prevalence of positive reactions to **Compositae** mixes and the SL mix in florists, corresponding to their occupational exposure, and confirmed that additional testing of SL mix will yield a notable share of contact allergies not detected with Compositae mixes, namely, 89 patients positive solely to SL mix in addition to 533 patients (also) reacting to a Compositae mix [269]. A Danish investigation concluded that SL mix is an indispensable, although insufficient, screening mixture in that country which may be safely supplemented with Compositae mix 2.5% and parthenolide in the TRUE Test™ system for screening, but, when Compositae sensitization is suspected, further extracts should be tested on the basis of the history [270].

Nevertheless, **extracts** are an important tool for individual diagnosis, too, and can be prepared from unusual elicitors of plant-ACD, such as *Hibiscus rosa-sinensis* L., which had been used in a Hindu ritual and sensitised the priest [271]. A Chinese patient had used a mixture of *Lysimachia clethroides* Duby, ethanol and borneol for the treatment of herpes zoster (as common in that region) and developed severe erythema multiforme-like ACD. Later, patch testing was positive to the mashed leaves 1:5 in pet. (negative in five volunteers) [272]. **Neem oil** is expressed from *Azadirachta indica* A. Juss. and popular as an ingredient of (Ayurvedic) skin care. A 32-year-old Italian with atopic eczema developed severe, oozing ACD of his face after applying a moisturising face pack containing neem oil and was found patch test positive to neem oil 10% pet. (10 healthy controls negative) [273]. Beyond the common, exotic flowers or greenery may cause ACD in gardeners or florists, such as *Eucalyptus* spp. in two Danish florists [274] or *Eucalyptus* spp. and *Tanacetum parthenium* (L.) Sch. Bip. in a 45-year-old Japanese florist [275].

A small, but important share of patients patch tested react to **topical herbal remedies**, such as 0.8% of patients in a highly specialised Belgian centre, where the most common allergens included *Myroxylon pereirae*, Compositae plants and tincture of benzoin. Of note, some patients did not react to the commercially available test allergens, but only to their own products [276]. Independently from exposure via herbal remedies, that is, including also sensitisation to the vital plant(s), 4% of 13,139 patients patch tested in Odense, Denmark, were allergic to Compositae, again confirming this family of plants being the most important sensitisers in Europe [277]. Interestingly, “biodynamic” or “organic” milk, but not conventionally processed milk, may contain traces of parthenolide, most likely owing to *Tanacetum vulgare* L. being grazed by the cattle; although the concentrations of around 0.05–0.07 ppm are low, they might suffice to elicit ACD by skin contact with milk in the most exquisitely sensitised [278].

**“Tulip dermatitis”** has been described and characterised regarding the aetiology by Dutch studies since the 1980s. As a consequence of globalisation and increasing demand, a large part of the global tulip flower production is now seen in India, namely, in the Kashmir valley. Screening 164 tulip-growing workers for dermatitis, 48 showed contact dermatitis, mostly of the hands, and 21 were contact allergic, mainly to *α*-methylene-*γ*-butyrolactone and/or an acetone extract of the tulips handled [279]. Plants are often treated with pesticides, which pose some risk of sensitisation, as demonstrated in a small sample of 30 Indian agricultural workers with work-related contact dermatitis, of whom 10 had positive reactions to thiurams (*n* = 4), propiconazole (*n* = 3) and metaldehyde (*n* = 2) [280].

**Propolis** is a resinous, aromatic smelling natural product produced by bees. It is mainly composed of the sticky coating of poplar buds, e.g., *Populus nigra* L., but also birch buds and buds from other trees are used by the bees. Owing to alleged fungicidal, anti-inflammatory and astringent effects, propolis is used in several “natural remedies”, drops, lozenges and tinctures, creams and ointments for the treatment of various conditions. Contact allergy is not rare (e.g., 2.34% among 28,474 patients patch tested 2009–2012 in the ESSCA network [281]) and can be quite severe. Allergic contact stomatitis has been described following use of propolis candies [282] and lozenges [283], pure propolis applied to the perinasal skin for cold relief [284], and topical and oral propolis for self-treatment of presumed herpetic lip infection [285], in the latter case accompanied by systemic allergic dermatitis. Moreover, patients contact allergic to yellow or white beeswax often react to propolis owing to contamination [286].

**Colophonium** is a by-product from the distillation of Pinaceae tree oil and an important contact allergen with 1.67% positive reactions in consecutively tested patients in Northeast Italy [287]. In the highly specialised Finnish Institute of Occupational Health, 118 of the patients assessed between 2002 and 2017 (4.6%) patch tested positive to colophonium. Most worked in the wood industry, as machinists, solderers, or in agriculture [288]. A “classical” source of colophonium exposure are the adhesives of plasters, as in the case of a 33-year-old professional football player who fixed his socks with these and developed severe ACD at his ankles [289]. A particular brand of test chambers (Finn Chamber™ AQUA) had used a colophonium derivative to fix filter paper to the chamber, which has caused two (reported) cases of positive test reactions to seemingly all allergens (the tackifier has subsequently been reformulated) [290,291]. Colophonium is used to treat violin and other string bows, but also elsewhere to increase grip, e.g., in a “liquid chalk” used for pole dancing, eliciting ACD of the hands in two young females sensitised to colophonium [292].

Exotic woods partly contain extremely potent sensitisers, as illustrated by a very severe allergic reaction with the clinical picture of Erythema multiforme major, which was induced by exposure to dust of pao santo/ferro (*Myroxylon scleroxylon* Tul.) [293]. ACD of exposed skin in a 53-year-old Brazilian furniture maker was caused by “peroba rosa” wood (*Aspidosperma polyneuron* Müll. Arg.) [294]. Japanese lacquer contains the very potent allergen urushiol (now available in the TRUE Test™ system [295]) capable of causing severe ACD [296]. Musical instruments are often made of exotic woods and may cause ACD to these in professional musicians, e.g., to palisander [297] or African blackwood (*Dalbergia melanoxylon* Guill. Perr.) [298]. Evidently, musical instrument makers can also be affected, as in the case of a 60-year-old Italian guitar maker sensitised to *Machaerium scleroxylon* Tul. [299]. Tars, e.g., from birch wood, have historically been used for topical treatment of various skin conditions, which has been abandoned for a long time owing to their carcinogenic potential. Hence they are rarely patch tested nowadays but have been the clue to the diagnosis in a 37-year-old Dutch male who enjoyed camping outdoors and wood-fires, but repeatedly suffered from facial dermatitis peaking 24 h after exposure—he was patch test-positive to a wood tar mix (12% pet.) [300].

### 3.9. New Allergens

Dermatologists—at least those who get involved in detective-like work-up of patients with suspected ACD and publish their well-documented results [301]—have an important role as sentinels in the detection of “new allergens”. This role can be augmented by networking in “cosmetovigilance” (as, e.g., the French REVIDAL/GERDA group), but can also be taken on in single offices and clinics. Future testing (and reports) will usually show whether a certain substance indeed poses a larger problem (and should be taken up in a series of commercially available test allergens)—see discussion below and Figure 1. The following Table 1 lists some findings regarding such new allergens, as reported in case reports or series during the period 2018/19.

## 4. Discussion

Contact allergy is a common condition [2]; see also [323]. A unique approach has been used in Ontario, Canada, to indirectly estimate the burden of ACD by using administrative reimbursement data for the patch test procedure. These data demonstrated an increasing use of this service over time (1992–2014) [324]. It may be speculated that the recent increase of non-occupational consultations may have, at least partly, been caused by the world-wide epidemic of MI contact allergy (see above). In some countries, limitations of the health system or health services offered may also limit the extent of patch testing. Consequently, data from these countries is scarce, e.g., from Nigeria [325], Romania [326], or remote areas of Brazil [327]. However, not only for a better global overview but—as written in the latter publication—to define preventive policies data analysis at the local level, such data are important [327]. While existing contact allergy may not necessarily lead to ACD if the allergen(s) can be sufficiently avoided, such avoidance often proves difficult. Difficulties increase with the ubiquity of substances, lack or shortcomings of identifying the presence of a substance in the environment [328] (cosmetic ingredient labelling is one laudable exception), the extent of cross-reactions with related substances, and a low individual elicitation threshold. In the following sections, some relevant public health aspects are discussed.

### 4.1. Allergen-Specific Aspects

Risk assessment aims at identifying risks regarding various toxicological endpoints, including sensitisation. Quantitative risk assessment (QRA) is a method still under development that has the objective of arriving at (presumably) safe exposure levels before the introduction of a substance onto the market, based on all relevant information at that point in time. Owing to problems mostly inherent to toxicological approaches such as inter-species differences, inter-individual variation and necessary safety margins, QRA will always rely on several assumptions, and decisions in terms of risk management based on QRA must always be monitored for effectiveness in the post-marketing period (see below). Nevertheless, QRA has undergone important development in the past years. It has, for instance, been recognised that “aggregate exposure” needs to be adequately taken into account, that is, exposure to one given compound by different products, e.g., to a preservative-like MI via cosmetic and household (and possibly occupational) products [329]. Presently, the fragrance industry has developed an improved version of quantitative risk assessment, even though several aspects have still been critically commented on by the Scientific Committee on Consumer Safety (SCCS) of the EU Commission (SCCS/1589/17; [330]). The health impact of potential policy measures aimed at reducing the concentration of the fragrance allergen geraniol in consumer products was analysed in a simulated population derived from multiple product use surveys. This exposure was estimated to result yearly in 34 new cases of geraniol contact allergy per million consumers in western and northern Europe, mainly due to exposure to household cleaning products. About twice as many consumers (60 per million) are projected to suffer from clinical symptoms due to re-exposure to geraniol. Policy measures restricting geraniol concentrations to <0.01% will noticeably reduce new cases of sensitisation and decrease the number of people with clinical symptoms as well as the frequency of occurrence of these clinical symptoms. According to the authors, the estimated numbers should be interpreted with caution and provide only a rough indication of the health impact [331]. Of note, the suggested use concentration restriction corresponds to a suggestion form the 2012 SCCS fragrance opinion for a set of allergens of concern (including geraniol), based on a more crude calculation (Table 13-5 in SCCS/1459/11; [93])).

Exposure to ingredients of **(oxidative) hair dyes** can lead to severe ACD in sensitised persons. From this background, attempts to screen consumers prior to a regular hair dye application for possible intolerance appear useful. Hitherto one industry-driven approach has been pursued, namely, the “self-test” or “allergy alert test” (AAT) using diverse methodologies [332]. While the latest protocol for the AAT [333] at least offers some degree of standardisation, and has shown good concordance between lay (consumer) and dermatological evaluation, several concerns remain, including, but not limited to, the added risk of sensitisation by application of an AAT prior to the hair dye product [334]. Screening for contact allergy by a suitable question(naire) may be a useful strategy, but its diagnostic validity must be assessed, ideally against sensitisation status as verified by a patch test. Along this line, US authors found the question “Do you get rashes when metal touches your skin?” to identify 77% of the nickel sensitised, while the positive (negative) predictive value was also satisfactory with 71% (84%) [335].

In cosmetics, exposure to **formaldehyde** is mostly via different formaldehyde releasers, which release varying amounts of free formaldehyde (and may be contact allergens in their own right). For formaldehyde-allergic patients, a declaration of free formaldehyde levels in products that may elicit ACD needs to be indicated by appropriate labelling of formaldehyde [137]. The detection of formaldehyde in products is possible with the chromotropic acid method; however, results do not necessarily correspond to HPLC analysis [336]. Cross-reactivity—that is, the sensitisation by one substance and elicitation also by other substance(s)—may pose unexpected problems, as illustrated by the case of a 57-year-old Spanish patient who developed face dermatitis following the use of a cream containing oxidised vitamin K (phytonadione epoxide). The use of this is still permitted, while non-oxidised vitamin K was prohibited in 2009 owing to the risk of ACD. Interestingly, the patient reacted to both oxidised and non-oxidised forms, the latter relevant for a rash 13 years earlier, making true cross-reactivity likely [337].

In the EU, the market authorisation of **bufexamac** was withdrawn almost 10 years ago by the European Medicines Agency (EMA) owing to an unacceptable incidence of severe ACD (and a largely lack of proof of efficacy). In contrast, in other countries such as Australia, bufexamac is still being marketed and still causes the same problems, as illustrated by a 41-year-old Australian patient whose ACD was so severe as to require hospitalisation [338]; the authors suggest reappraisal of marketing authorisation in the countries outside the EU. While special allergens such as bufexamac may cause severe ACD, they are easily avoidable, which does not hold true for ubiquitously occurring allergens such as metals, preservatives (biocides) or fragrances. This entails a chronic or relapsing course of ACD, which may considerably impair QoL, as illustrated in fragrance-allergic patients [339].

Ingredients of cosmetics and indeed all products coming into contact with the skin may exert various unwanted effects beyond sensitisation risk. In the case of **iodopropynyl butylcarbamate** (IPBC), it is iodine liberation and systemic availability that led the EU to limit the use concentration to 0.02% in rinse-off, 0.01% in leave-on cosmetics and 0.0075% in deodorants, see (EC) No 1223/2009. The recent observation of frequent concomitant allergic reactions to IPBC and iodine [340] supports this cautionary approach, pointing to a relevant co-exposure to both IPBC, which is a contact allergen in its own right [341], and (liberated) iodine.

### 4.2. Pre-Marketing Screening: Alternative Methods

In terms of primary prevention, hazard assessment (in this context: sensitising potency) is a crucial starting point for risk assessment and management aiming at the prevention of new cases of sensitisation, that is, primary prevention. An identified sensitisation hazard will trigger labelling and hazard warnings according to the Globally Harmonized System (GHS), implemented in the EU as Classification, Labelling and Packaging Regulation EC 1272/2008; the criteria used for this purpose have been discussed recently [342]. Besides hazard information, restrictions in maximum permitted use levels are intended to serve primary prevention. Evidently, correct identification of the hazard and adequate exposure assessment are crucial for risk management to yield maximum concentration levels, which actually turn out to be reasonably safe. Failures in this regard have resulted in several epidemics of contact allergy, the last one following the use of methylisothiazolinone (MI) in cosmetics (Section 3.3).

The implementation of European Union regulations such as REACH (Registration, Evaluation, Authorisation and Restriction of Chemicals) and the Cosmetics Regulation have generated considerable effort to develop non-animal methods to assess skin sensitisation. The adverse outcome pathway (AOP) for skin sensitisation, published by the Organisation for Economic Cooperation and Development (OECD) in 2012 [343,344], has been the basis for new alternative methods addressing specific key events (KEs) of this AOP. So far, 3 KEs are covered by OECD validated test guidelines: The direct peptide reactivity assay (DPRA) addressing the protein reactivity (KE1) [345], the ARE-Nrf2 Luciferase test method addressing the keratinocytes activation (KE2) [346] and the human cell line activation test (H-Clat), the U937 cell line activation test (U-SENS) and the interleukin-8 reporter gene assay (IL-8 Luc Assay) addressing antigen-presenting cells activation (KE3) [347]. Although these methods are individually performing quite well to identify potential skin sensitisers, they only address one specific KE. Moreover, they show some limitations in addressing bioavailability, metabolic activation and danger signals. Therefore, several new approaches are currently under development based on reconstructed human epidermis (RHE) models such as the SENS-IS assay based on the expression analysis of a large panel of genes relevant to the sensitisation process [348] or based on co-culture systems such as the HaCaT/THP1 CoCulture Activation Test (COCAT), taking advantage of interactions/cross-talk between keratinocytes and THP1 cells [349]. In parallel, considerable work on the development of integrated approaches to testing and assessment (IATA) aimed at the definition of integrated test strategies (ITS) has been initiated at the OECD level [350]. Several options have thus been proposed and are currently under evaluation.

### 4.3. Post-Marketing Surveillance

Given the repeated observation of new allergens appearing either in terms of sporadic cases or sometimes amounting to a true epidemic, as in the case of MI, failures of pre-marketing risk assessment and subsequent risk management are regularly occurring. Alternative methods for qualitative assessment have been validated and in-chemico approaches are in the process of validation (see above). However, inevitably, as also in the time when animal experiments were used, false-negative or “false-low” results yielded too high use concentrations in products containing these substances. Contact allergy in humans will thus probably follow, which needs to be picked up early by post-marketing surveillance. Often, the first alert of a possible problem is a case report documenting one or a few, often highly sensitised, patients. It is of importance that such case reports are actually published and that these reports provide a basic level of quality [301]. A second or a third such case report may confirm the initial observation and add more evidence regarding appropriate test methods for the newly identified contact allergen (test concentration/vehicle). Finally, systematic testing by inclusion in a special series, tailored to the exposure(s) the new allergen has been encountered in, will give a well-substantiated impression of the importance of a new allergen, and enable the diagnosis of specific contact allergy to this even in cases where it was not initially suspected to be an allergen [6], as illustrated in Figure 1, that is, case-by-case. The body of clinical evidence, provided by patch test studies hitherto published, must regularly be appraised to identify adjustment to risk management possibly necessary [342]. To this end, the SCCS of the EU Commission and similar bodies provide important guidance.

### 4.4. Implications for Regulation

The negative implications of insufficient information on ingredients of virtually all but cosmetic products are a regularly recurring issue in the literature. For instance, glove constituents are not declared, which creates an unacceptable risk for those sensitised to suffer from ACD by hidden sources of allergens [351]. The provision of accelerator-free gloves for all health care workers would be a theoretical alternative [179]. Unfortunately, owing to high(er) costs, this is a costly alternative, compared with the simpler option of having a full declaration of ingredients (and, independently, as low as possible residual levels of these). Unfortunately, the newly revised EU (in vitro) Medical Devices Regulation (EU 2017/745 and EU 2017/746) provide no benefit for the management of ACD, because they do not mention any need for information on the qualitative composition nor labelling on the packaging of medical devices [228]. Many delays could be avoided and public resources saved if a stringent, comprehensive mechanism could be implemented offering full information on the ingredients of all types of products.

Once the regulation has become effective, and the window has passed when pre-regulation exposure levels are still permissible in products already on the shelf for sale, exposure to an allergen may still not be eliminated entirely. As one rather self-evident, but possibly often overlooked loophole, a Finnish author described the case of a 27-year-old man with chronic, severe foot dermatitis which was elicited by application of a certain brand of moisturiser which had long been withdrawn, but had lurked in the patient’s cupboard for several years [352]. It is interesting to note that even although ingredients have been banned or restricted in some areas of the world, usually with more or less immediate beneficial effects in terms of reducing the incidence of sensitisation, and most likely also elicitation of ACD in those sensitised, they are continued to be used in other areas of the world, such as bufexamac (see above) and also methyldibromo glutaronitrile, banned in the EU more than 10 years ago, but being the second most common allergen in Nigeria [325]. However, owing to the complexities of national independence, commercial interests and market laws, and imbalances across the globe, any global regulation of substances appears utopic.

## 5. Conclusions

Living in the “information age” of the Anthropocene, we are flooded by information via digital formats—but this information is often totally useless for our lives, private or professional. On the other hand, relevant information is often missing or difficult to filter out. In the present review, we have tried to compile relevant, current, mostly clinical information for the field of contact allergy and allergic contact dermatitis, respectively. The aim is to provide an overview of on-going topics mainly for clinicians, but also for others interested in the field (e.g., regulators, industry) largely according to the objective of “evidence based medicine” to periodically compile a critical summary. Further, we have addressed some repeated observations which point to systematic shortcomings in the flow of information, particularly from producers and distributors to clients, patients, and their clinicians, which is most acute in the field of medical devices. We see the requirement of better exploiting the potential of the “information age” to achieve something which could also be called transparency, for the benefit of those struggling to escape the consequences of contact allergy.

## Figures and Tables

**Table 1:** New allergens reported 2018–2019. F, female; M, male; age in years.

Case(s)	Exposure	Allergen	Ref.
M (55)	Water-based metalworking fluid	2-Amino-2-methyl-1-propanol (2% pet.)	[302]
F (33)	“Hypoallergenic cosmetic product for sensitive skin”	Bakuchiol (0.1% pet.)	[303]
F (67)	Home-grown *Cannabis sativa* and *C. indica*	Positive to entire dried flower (10 controls negative)	[304]
M (50)	Body lotion	Capryloyl glycine (1% aq./ethanol 1:1)	[305]
F (51)	Brand hand and foot cream, respectively	Sodium cetearyl sulfate (10% pet.)	[306]
M (47)	Cream deodorant	Cetyl PEG/PPG-10/1 dimethicone (copolymer) 5% pet.	[307]
3 M, (35–60)	Skin protection cream	Cocoamphopropionate (1% aq.)	[308]
F (36)	“Label dermatitis” due to hot melt adhesive	dimethyl (p-methoxybenzylidene)malonate (2% pet.)	[309]
F (34)	Multicoloured bikini and black and white dress	Disperse Blue 360 tested on polyester fabrich swatches	[310]
M (79)	Eyedrops … and wife’s face cream	Face cream “as is” and ingredient hydroxyacetophenone 0.6% aq.	[311]
M (21)	Moisturising cream	10-hydroxydecenoic acid	[312]
M (53,58)	Production of heat-sensitive polyethylene terephthalate films	*N*-(4-hydroxyphenyl)benzenesulfonamide	[313]
F (43)	Face cream	Isopropyl lauroyl sarcosinate (5% ethanol)	[314]
F (55, 59,92)	Antimicrobial ointments/bandage	Isopropyl methylphenol (0.001–1.5% pet.)	[315]
F (20,51), M (55)	Lip balm causing cheilitis	Lauryl PCA (20% pet.)	[316]
M (10)	Non-woven polyethylene sheets used during surgery	Octadecyl 3-(3,5-di-tert-butyl-4-hydroxyphenyl)propionate (Irganox 1076) (0.05–2% pet.)	[317]
F (66), M (65)	Perioperative antiseptic	Olanexidine gluconate (“as is”); excipients negative	[318]
F (33)	Moisturizing cream for atopic skin	Polyacrylamide/C13-4 isoparaffin/laureth-7 mix (tested “as is”)	[319]
F (26)	“Rosa mosqueta” oil	Positive ROAT with product (*R. canina* fruit oil; *R. rubiginosa* seed oil), negative to tocopherol (antioxidant in product)	[320]
4 F (26–52)	Brand deodorant	Sorbitan caprylate (1% pet.)	[321]
M (46)	Brand sunscreen	Vinylpyrrolidone/eicosene copolymer 5% pet.	[322]

## Abbreviations

**Shorthand**	**Explanation**
ACD	Allergic contact dermatitis
CI	Confidence interval
EBS	European baseline series
ECHA	European Chemicals Agency (echa.europa.eu)
EECDRG	European Environmental and Contact Dermatitis Research Group (<www.eecdrg.org>)
ESCD	European Society of Contact Dermatitis (<www.escd.org>)
FM	Fragrance mix
GC-MS	Gas chromatography-mass spectrometry
HICC	Hydroxyisohexyl 3-cyclohexene carboxaldehyde
HPLC	High-performance liquid chromatography
ICPMS	Inductively coupled plasma mass spetrometry
IVDK	Information Network of Departments of Dermatology (<www.ivdk.org>)
LTT	Lymphocyte Transformation Test
M(C)I	Methyl(chloro)isothiazolinone
OOHs	Hydroperoxides
OR	Odds ratio
pet.	Petrolatum (as patch test vehicle)
QoL	Quality of life
ROAT	Repeated open application test
